# Sleep-time physiological recovery is associated with eating habits in distressed working-age Finns with overweight: secondary analysis of a randomised controlled trial

**DOI:** 10.1186/s12995-021-00310-6

**Published:** 2021-06-28

**Authors:** Elina Järvelä-Reijonen, Suvi Järvinen, Leila Karhunen, Tiina Föhr, Tero Myllymäki, Essi Sairanen, Sanni Lindroos, Katri Peuhkuri, Maarit Hallikainen, Jussi Pihlajamäki, Sampsa Puttonen, Riitta Korpela, Miikka Ermes, Raimo Lappalainen, Urho M. Kujala, Marjukka Kolehmainen, Jaana Laitinen

**Affiliations:** 1 Institute of Public Health and Clinical Nutrition, Clinical Nutrition, University of Eastern Finland, P.O. Box 1627, FI-70211 Kuopio, Finland; 2 Institute of Clinical Medicine and Clinical Nutrition, Kuopio University Hospital, P.O. Box 100, FI-70029 KYS Kuopio, Finland; 3 Faculty of Sport and Health Sciences, University of Jyväskylä, P.O. Box 35, FI-40014 Jyväskylä, Finland; 4 Department of Psychology, University of Jyväskylä, P.O. Box 35, FI-40014 Jyväskylä, Finland; 5 Department of Psychology, Karlstad University, SE-651 88 Karlstad, Sweden; 6 Medical Faculty, Pharmacology, Medical Nutrition Physiology, University of Helsinki, P.O. Box 63, FI-00014 Helsinki, Finland; 7 Social Services and Health Care, City of Helsinki, Helsinki, Finland; 8 Finnish Institute of Occupational Health, P.O. Box 40, FI-00251 Helsinki, Finland; 9 Medical Faculty, Human Microbe Research Program, University of Helsinki, P.O. Box 63, FI-00014 Helsinki, Finland

**Keywords:** Dietary behaviour, Heart rate variability, Intuitive eating, Parasympathetic activity, Stress

## Abstract

**Background:**

Association of physiological recovery with nutrition has scarcely been studied. We investigated whether physiological recovery during sleep relates to eating habits, i.e., eating behaviour and diet quality.

**Methods:**

Cross-sectional baseline analysis of psychologically distressed adults with overweight (*N* = 252) participating in a lifestyle intervention study in three Finnish cities. Recovery measures were based on sleep-time heart rate variability (HRV) measured for 3 consecutive nights. Measures derived from HRV were 1) RMSSD (Root Mean Square of the Successive Differences) indicating the parasympathetic activation of the autonomic nervous system and 2) Stress Balance (SB) indicating the temporal ratio of recovery to stress. Eating behaviour was measured with questionnaires (Intuitive Eating Scale, Three-Factor Eating Questionnaire, Health and Taste Attitude Scales, ecSatter Inventory™). Diet quality was quantified using questionnaires (Index of Diet Quality, Alcohol Use Disorders Identification Test Consumption) and 48-h dietary recall.

**Results:**

Participants with best RMSSD reported less intuitive eating (*p* = 0.019) and less eating for physical rather than emotional reasons (*p* = 0.010) compared to those with poorest RMSSD; participants with good SB reported less unconditional permission to eat (*p* = 0.008), higher fibre intake (*p* = 0.028), higher diet quality (*p* = 0.001), and lower alcohol consumption (*p* < 0.001) compared to those with poor SB, although effect sizes were small. In subgroup analyses among participants who reported working regular daytime hours (*n* = 216), only the associations of SB with diet quality and alcohol consumption remained significant.

**Conclusions:**

Better nocturnal recovery showed associations with better diet quality, lower alcohol consumption and possibly lower intuitive eating. In future lifestyle interventions and clinical practice, it is important to acknowledge sleep-time recovery as one possible factor linked with eating habits.

**Trial registration:**

ClinicalTrials.gov Identifier NCT01738256, Registered 17 August 2012.

**Supplementary Information:**

The online version contains supplementary material available at 10.1186/s12995-021-00310-6.

## Introduction

Obesity, psychological distress, and perception of inadequate sleep are alarmingly common among working-age adults [1–3]. As all these factors predispose to negative health outcomes, it is important to examine factors that support health in this high-risk population. One factor that has a major influence on our health and well-being is sufficient recovery [4–7]. Recovery may affect health through various pathways, including eating habits (i.e., diet and eating behaviour).

Recovery from stress comprises two interlinked phenomena: 1) psychological recovery experiences which include psychological detachment from the stressor (e.g., work), relaxation, mastery and control [8]; and 2) physiological recovery. Physiological recovery refers to a process during which the body unwinds from daily strain. This process stems from the physiological systems of the body, such as the autonomic nervous system and the cardiovascular and metabolic systems, that are constantly functioning towards maintaining homoeostasis in the face of changing environmental stressors [9]. The functioning of these physiological systems can be measured noninvasively and objectively with heart rate variability (HRV) measurement [5–7, 10, 11]. HRV reflects the balance between the parasympathetic and sympathetic activity of the autonomic nervous system [12]. In stress response, the sympathetic nervous system is predominant, parasympathetic activation is low, heart rate is increased, and HRV is low [13, 14]. While the sympathetic nervous system is responsible for the “fight or flight” state of the body, the parasympathetic nervous system is predominant at rest [14]. Thus, during recovery, parasympathetic activation dominates, heart rate is decreased, and HRV is high [13].

The widely recognised association between subjective stress and unhealthy eating habits – such as increased emotional and uncontrolled eating [15, 16], decreased intuitive eating and eating competence [15], and poor quality of diet [17, 18] – raises the question whether HRV, a physiological marker of recovery and stress, shows parallel associations with eating habits. A recent study found that poorer self-assessed ability to recover and sleep problems were linked to more frequently choosing unhealthy foods [19]. Low HRV has been linked to subjective loss-of-control eating [20, 21] and low diet quality [21]. High HRV has been reported to be associated with stronger regulation of food cravings [22] and of eating behaviour [23]. Meule et al. [24] found low HRV at rest to be associated with restrained eating, i.e., restriction of food intake to control weight, whereas Finch et al. [25] reported no differences in HRV between those who had eaten healthy/unhealthy comfort foods vs. no food after a social stress test. In these previous studies, the participants’ BMI has ranged from underweight to obesity, which can be considered a marked confounder in terms of both HRV and eating habits. To the best of our knowledge, there is only one study where the authors have compared both HRV and eating response between obese and non-obese participants [26]. This study reported no differences in HRV during either positive or negative mood induction phase, but the high-energy food introduced after the mood inductions (especially positive) elicited a higher HRV among the obese group [26].

Associations of a single dietary component (e.g., fat or sodium intake, nut consumption) with HRV have been reported in several studies; as reviewed by Young and Benton [27], the current evidence points towards an association between healthy aspects of diet and higher HRV. In line with this, a recent observational study by Reginato et al. [28] reported a positive association between fruit consumption and HRV. Considering diet more broadly, the Mediterranean diet has been shown to associate with higher HRV [29]. HRV has been considered a promising marker for identifying the influence of diet on HRV, although the possibility of a two-way interaction between diet and HRV has also been acknowledged [27].

Only one study so far has examined the association between night-time HRV and eating habits: Jaatinen et al. [30] found a positive effect of 4-week daily ingestion of dietary component (yoghurt with bioactive component) on nocturnal HRV in participants with anxiety. The lack of studies addressing the possible link between night-time HRV and eating habits is surprising given the importance of sleep in the recovery process [5–7]. After all, studies examining sleep and eating habits point to a multifactorial effect of short sleep duration on consumption of energy-dense, highly palatable foods [31]. A recent study found that poorer self-assessed ability to recover and sleep problems were both linked to more frequently choosing unhealthy foods [19]. Further, in cross-sectional studies, poor sleep quality has been associated with unbeneficial eating-behaviour traits, such as emotional eating and uncontrolled eating [32].

Besides the lack of nocturnal HRV measurements, a further two important gaps in the current literature on HRV and eating habits need to be noted. First, no earlier study populations have comprised both chronically stressed and overweight/obese participants. Second, there is clear need for HRV measurements spanning several hours and days. The majority of the previous studies on the association between HRV and eating habits have been conducted in laboratory settings over short time periods [21–24, 33]. Due to the ever-changing status quo between the sympathetic and parasympathetic nervous system, monitoring HRV over a longer period in free-living circumstances would provide more reliable measures of the autonomic nervous system functioning.

To address the current research gaps, our aim was to explore among working-age individuals with psychological distress and overweight whether any associations exist between physiological recovery measured in real-life conditions over several nights and eating habits. Due to the importance of sleep in the recovery process [5–7], we focused on the association of sleep-time HRV-based recovery measures with eating habits covering both eating behaviour and diet quality. To the best of our knowledge, this study is the first to investigate these associations in a free-living, non-clinical working-age population at high risk of stress-related negative health outcomes.

## Methods

### Participants and study design

Our data consist of baseline measurements of the Elixir randomised controlled trial conducted in the cities of Kuopio, Jyväskylä, and Helsinki, Finland. The aim of the Elixir study was to investigate the effects of acceptance and commitment therapy (ACT) and cognitive behavioural therapy (CBT) on metabolic syndrome risk factors, psychological measures, and general well-being [34]. Participant recruitment occurred in two phases between August 2012 and January 2013 via advertisements in local newspapers. Participant eligibility assessment utilised telephone interviews.

Participants were randomised into four groups: (1) ACT-based intervention via six face-to-face group sessions led by a psychologist, (2) ACT-based mobile-phone-application intervention following one introductory session, (3) CBT-based intervention carried out on the Internet with no group sessions, and (4) a control group attending only the measurement sessions.

Inclusion criteria were age of 25–60 years, BMI of 27–34.9 kg/m^2^, psychological distress (≥3/12 points on the General Health Questionnaire, GHQ-12 [35]), and having a computer with Internet access. Exclusion criteria included three-shift and night work, diagnosed severe chronic illnesses, and having a pacemaker [34]. In the present analyses, additional exclusion criteria were missing recovery data (RMSSD and/or Stress Balance) (*n* = 16) and current or previous use, or missing data on the use, of α- or β-adrenergic blocking agents affecting HRV (*n* = 30) [36]. The present study involved 252 (83% female) participants.

### Measures

#### Recovery

To collect continuous beat-to-beat R-R interval data in real-life settings, each participant was instructed to wear a Bodyguard device (Firstbeat Technologies Ltd., Jyväskylä, Finland) attached with two electrodes on the chest for three consecutive days and nights. The participants reported in the study diary the time they went to bed and their sleeping time. From these data, HRV-based nocturnal recovery measures were derived using the Firstbeat Analysis Server software (v 5.3.0.4). The software first detects and corrects falsely detected, missed, and premature beats, i.e., artefacts [37, 38]. HRV data from all successfully recorded nights (percentage of artefacts ≤15%) were used in the analyses. The majority (81%) of the successful recordings spanned 3 nights (range 1–4 nights).

In the R-R interval data, the stress state consists of elevated individual HR, decreased HRV, and low respiration rate compared to HR. Recovery is detected when parasympathetic (vagal) activation predominates the autonomic nervous system. In the R-R interval data, the recovery state consists of low individual HR accompanied by great and uniform HRV [37].

The sleep-time recovery measures used were mean values of the root mean square of successive differences (RMSSD) and Stress Balance (SB) over the period of the 1–4 recording days. RMSSD is a commonly used and reliable time-domain measure of HRV [12, 39, 40]. Higher RMSSD indicates increased parasympathetic activity and better recovery [39, 40]. SB indicates the temporal ratio of recovery to stress reactions during self-reported sleep periods [10]. Possible numerical values of SB range from − 1 to 1 with values from − 1 to 0 indicating weak recovery, values from 0 to 0.5 moderate recovery, and values from 0.5 to 1 good recovery [10].

#### Eating habits: eating behaviour and diet quality

The 21-item Intuitive Eating Scale, IES [41] measured intuitive eating. Cronbach’s α was 0.79 for the entire scale and 0.69; 0.86; 0.76 for the subscales *Unconditional permission to eat*, *Eating for physical rather than emotional reasons*, and *Reliance on internal hunger/satiety cues*, respectively.

The Three-Factor Eating Questionnaire, TFEQ-R18 [42], measured *Cognitive restraint*, *Uncontrolled eating*, and *Emotional eating*. Cronbach’s αs were 0.68; 0.87; and 0.88 for the scales, respectively.

Of the Finnish Health and Taste Attitude Scales, HTAS [43], we used the subscales *Pleasure* and *Using food as a reward* (Cronbach’s αs 0.72 and 0.78, respectively).

Eating competence was measured using preliminary Finnish translation of ecSatter Inventory 2.0 (ecSI 2.0™) [44]. Cronbach’s α was 0.76 for the entire scale and 0.60; 0.65; 0.59; 0.76 for the subscales *Eating attitudes*, *Food acceptance*, *Internal regulation*, and *Contextual skills*, respectively.

The Index of Diet Quality (IDQ) questionnaire [45] measured adherence to Nordic and Finnish nutrition recommendations. Alcohol consumption during the previous 6 months was measured using Alcohol Use Disorders Identification Test Consumption, AUDIT-C [46] (Cronbach’s α 0.69).

As described previously, 48-h dietary recall was conducted on the telephone between Tuesday and Friday [15]. Nutrient intake was calculated using AivoDiet software (v 2.0.2.2, Aivo Ltd., Turku, Finland) utilising the Fineli® Finnish Food Composition Database (National Institute for Health and Welfare, Nutrition Unit, Helsinki, Finland). To assess diet quality, we used energy intake (kcal), energy nutrient intake (protein, fat, saturated fat, monounsaturated fat, polyunsaturated fat, carbohydrate, and sucrose as percentage of energy intake, E%), and fibre intake (g/MJ).

#### Background variables

Background information, e.g., gender, education level, marital status, and work situation, was collected with a questionnaire. BMI (kg/m^2^) was based on measured height and weight [34]. The 14-item Perceived Stress Scale (PSS) measured the degree of perceived stress in life [47] (Cronbach’s α 0.88). Perceived work ability was measured with the 11-item Work Ability Index (WAI) questionnaire [48] (Cronbach’s α 0.75). Total sleep time (mean) was calculated based on the self-reported sleep times marked in the sleep diary.

### Statistical analysis

Data are presented as means and standard deviations (SDs) unless otherwise stated. Statistical analyses were performed using IBM SPSS statistics version 25 or newer (SPSS Inc., Chicago, IL). Two-tailed *p* value < 0.05 characterised statistical significance. We used Pearson’s correlation coefficient in correlation analyses. In ANOVA and ANCOVA analyses described below, we used standardised residual histograms to assess normality and, when necessary, performed logarithmic or square root transformations to achieve normally distributed residuals. Effect sizes were estimated with partial η^2^.

Considering that the association between parasympathetic activity and health appears to be nonlinear [49] and to compare participants having differing recovery levels, we categorised the participants into three categories for both recovery measures. SB groups were formed based on the predetermined cut-off values [10] dividing the participants into groups of weak (− 1–0, *n* = 59, 23.4%), moderate (0–0.5, *n* = 87, 34.5%), and good recovery (0.5–1, *n* = 106, 42.1%). For RMSSD, no cut-off values exist, so the participants were split into tertiles (*n* = 84 each) at values 25.00 and 37.30. The participants with the highest RMSSD had the best recovery, while the participants in the other two tertiles had average and the weakest recovery based on the RMSSD values in this population.

To explore the main effects of recovery independently from the multicentre and -phase study design, we ran 3x3x2 three-way ANOVAs with the recovery group as the variable of interest, adjusting for study centre and starting time of the study (autumn/spring). After these main analyses, we then ran 3x3x2x2 ANCOVAs with the recovery group as the variable of interest, adjusting for study centre, starting time of the study, gender, and BMI (kg/m^2^). Eating habit-related variables were treated as dependent variables in both sets of analysis. As follow-up tests, we used Šidák-corrected post hoc comparisons. Only group comparisons with significant main effects of recovery group are reported in this article. The main effects of group for all outcome variables are reported in Additional file.

To investigate the possible effect of worktime on the results, we ran the analyses also among the subgroup of participants who reported working regular daytime hours (*n* = 216).

## Results

### Participants

Descriptive participant characteristics are shown in Table 1. The participants were middle-aged on average, and most had either moderate or good work ability. The participants’ RMSSD and Stress Balance (SB) had a moderate positive correlation (r = 0.316, *p* < 0.001). Total sleep time (*n* = 238) had a weak, non-significant correlation with RMSSD (r = 0.073, *p* = 0.260) and a weak yet significant correlation with SB (r = 0.145, *p* = 0.025). The majority of the participants had at least college education (78%) and were married or cohabitating (73%). The currently employed participants (96%) as well as those outside of workforce (4%) reported having either daytime work (86%), two-shift work (6%), or other irregular daytime work (9%). The majority perceived their work as mentally somewhat, rather, or very strenuous (94.4% altogether), while the most common assessments of physical work strain were ‘rather easy’ and ‘not at all strenuous’ (71.8% altogether). The majority (89%) reported attempting to lose weight.

**Table 1:** Descriptive characteristics of the participants (*N* = 252)

	Mean (SD)	Range (possible range)	N	%
Age (years)	48 (8)	27–61		
BMI (kg/m^2^)	31.3 (3.1)	25.3–40.1		
RMSSD	34.2 (16.6)	6.8–128.0		
Stress Balance	0.32 (0.48)	−1.0–1.0 (−1.0–1.0)		
Perceived stress (PSS score)	26.4 (7.6)	7.0–52.0 (0.0–56.0)		
Work ability (WAI score)	35.7 (5.6)	10.5–46.0 (7.0–49.0)		
Poor (7–27)			17	6.7
Moderate (28–36)			102	40.5
Good (37–43)			124	49.2
Excellent (44–49)			9	3.6

*BMI* Body Mass Index, *RMSSD* Root Mean Square of the Successive Differences, *PSS* Perceived Stress Scale [47], *WAI* Work Ability Index [48]

Among the RMSSD and SB categories, no significant differences emerged in the continuous variables age, BMI, perceived stress, and work ability except for significant difference in age and mean WAI score between the RMSSD tertiles. The mean age of the RMSSD tertile with best recovery (44.3 years, SD 8.1 years) was lower compared to the tertiles with average (49.6 years, SD 7.0 years, *p* < 0.001) and weakest recovery (50.5 years, SD 6.4 years, *p* < 0.001), F(2,249) = 18.129, *p* < 0.001 (Šidák post hoc). The mean WAI score in the RMSSD tertile with best recovery (37.0, SD 5.0) was higher compared to the tertile with weakest recovery (34.5, SD 5.8), F(2,249) = 4.288, *p* = 0.015.

### Eating habits in the recovery groups

#### Eating behaviour

**Fig. 1:**
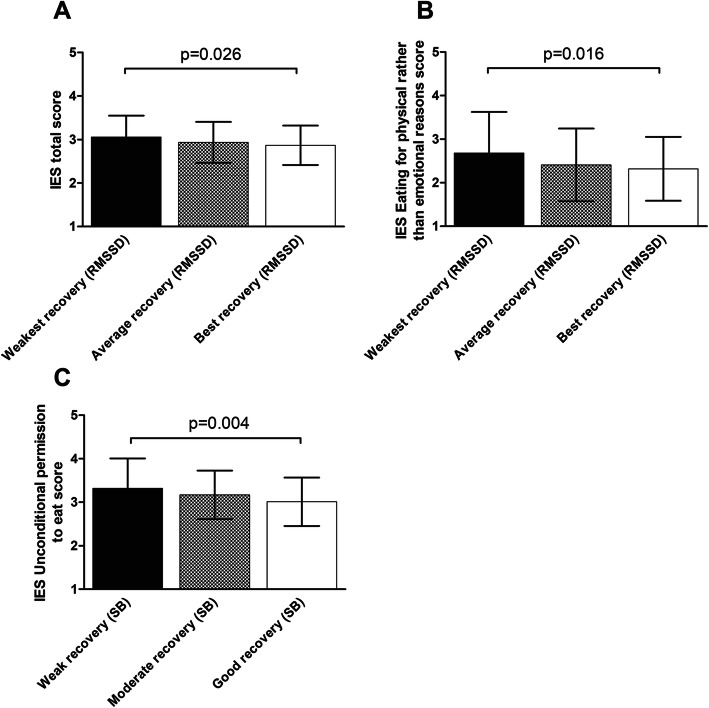
Differences among RMSSD tertiles (**A**, **B**) and Stress Balance groups (**C**) in eating-behaviour variables. Bars represent unadjusted means and SDs; *p* values are based on three-way ANOVAs with Šidák post hoc comparisons. Independent variables: RMSSD tertile / Stress Balance group, study centre, starting time of the study. [IES=Intuitive Eating Scale; RMSSD = Root Mean Square of the Successive Differences; SB=Stress Balance]

The RMSSD tertile with best sleep-time recovery ate less intuitively (IES total score) than the tertile with weakest recovery (partial η^2^ = 0.032; *p* = 0.026, pairwise Šidák post hoc comparison) (Fig. 1A). The RMSSD tertile with weakest recovery scored higher on *Eating for physical rather than emotional reasons* (a subcomponent of IES) than the tertile with best recovery (partial η^2^ = 0.037; pairwise *p* = 0.016) (Fig. 1B). The group main effects remained significant after including gender and BMI (Additional file, Table 1). In this secondary analysis, the RMSSD tertile with weakest recovery scored significantly higher on *Eating for physical rather than emotional reasons* than the tertile with average recovery (partial η^2^ = 0.045; pairwise *p* = 0.030). In the subgroup analyses involving only those working regular daytime hours, the main effect of RMSSD tertile on IES total score was no longer significant. The main effect of RMSSD tertile on *Eating for physical rather than emotional reasons* remained borderline significant in the secondary analysis (*p* = 0.052), but not in the main analysis.

Participants with good sleep-time recovery as measured by SB (i.e., most recovery reactions in relation to stress reactions) exhibited stronger avoidance towards eating freely whatever desired (*Unconditional permission to eat*, a subcomponent of IES) than participants with weak recovery (partial η^2^ = 0.038; pairwise *p* = 0.004) (Fig. 1C), a result that remained in the secondary analysis (Additional file, Table 2). In addition, only after adding gender and BMI to the model was the group main effect in intuitive eating (IES total score) significant (partial η^2^ = 0.026; main effect *p* = 0.039). The post hoc tests showed a non-significant tendency (pairwise *p* = 0.068) for participants with good recovery to report less intuitive eating (IES total score) compared with participants with weak SB recovery. In the subgroup analyses, the main effect of SB group on *Unconditional permission to eat* score remained in the secondary analysis (partial η^2^ = 0.037, pairwise *p* = 0.021), but not in the main analysis; also, SB group had no significant main effect on intuitive eating (IES).

#### Diet quality

Among the RMSSD tertiles (Additional file, Table 3), a significant between-group difference was observed in AUDIT-C scores only after including gender and BMI. The tertile with weakest recovery scored higher than the tertile with average recovery (partial η^2^ = 0.029; pairwise *p* = 0.028). In the subgroup analysis, the main effect of RMSSD tertile on AUDIT-C score remained borderline significant (partial η^2^ = 0.028, main effect *p* = 0.053), but only in secondary analysis.

**Fig. 2:**
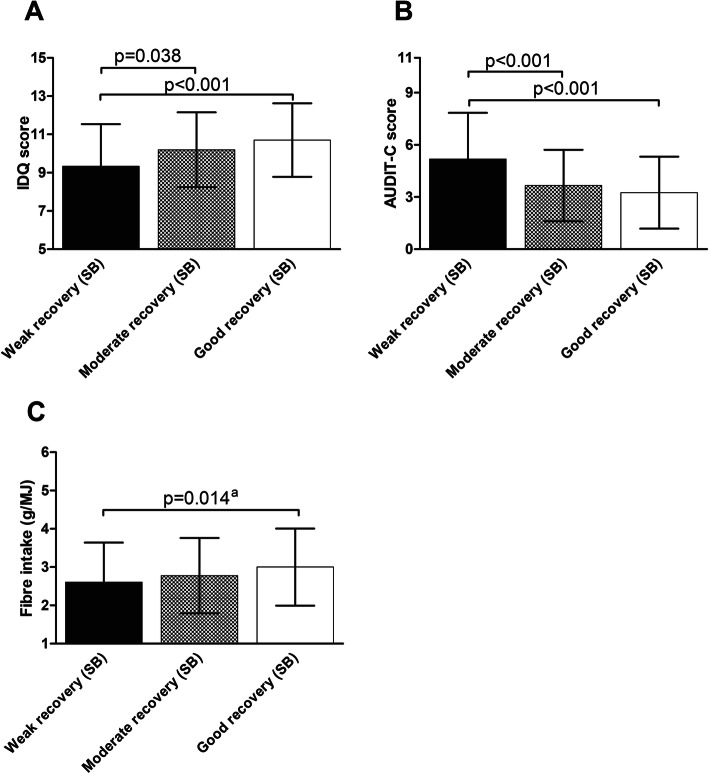
Differences among Stress Balance groups in diet-quality variables. Bars represent unadjusted means and SDs; *p* values are based on three-way ANOVAs with Šidák post hoc comparisons. Independent variables: Stress Balance group, study centre, starting time of the study. IDQ = Index of Diet Quality; AUDIT-C = Alcohol Use Disorders Identification Test Consumption; SB=Stress Balance. ^a^*p* value based on the LN transformed fibre intake (g/MJ)

Participants with weak recovery as measured by SB had lower diet quality (IDQ scores) than those with good (partial η^2^ = 0.055; pairwise *p* < 0.001) and moderate recovery (pairwise *p* = 0.038) (Fig. 2A). Further, groups with moderate and good recovery had lower alcohol consumption (AUDIT-C scores) compared with participants with weak recovery (partial η^2^ = 0.099; pairwise p < 0.001, both comparisons) (Fig. 2B). The participants with good recovery had higher fibre intake (g/MJ, LN transformed variable) than participants with weak recovery (partial η^2^ = 0.029; pairwise *p* = 0.014) (Fig. 2C, Additional file, Table 4). The results for AUDIT-C scores and fibre intake remained in the secondary analyses (Additional file, Tables 3 and 4), while IDQ score only differed between participants with weak and good recovery. In the subgroup analyses, the results for AUDIT-C scores remained, IDQ score difference remained between participants with weak and good recovery, and the main effect of fibre intake disappeared in both primary and secondary analyses.

## Discussion

The present study is the first to explore whether physiological recovery measured during several consecutive nights is related to eating habits in stressed adults with overweight or obesity. We investigated how physiological, heart rate variability (HRV)-based sleep-time recovery is associated with eating habits, covering both eating behaviour and diet quality, in a real-life setting. Our results suggest that in a working-age population with psychological distress and overweight, higher sleep-time parasympathetic activity–indicating better physiological recovery–associates with more health-promoting diet quality, lower alcohol consumption and possibly less intuitive eating.

Better sleep-time recovery, measured by the ratio between stress and recovery during sleep (Stress Balance), was significantly associated with higher fibre intake and higher overall diet quality, supporting previous–although scarce–literature. Higher HRV measured in a laboratory setting has been associated with health-promoting features of diet, such as higher intake of green leafy vegetables [33] and fruit [28], adherence to the Mediterranean diet [29], and higher diet quality [21]. In our study, weaker recovery, measured by both RMSSD and Stress Balance, was also associated with higher reported long-term alcohol consumption. This supports earlier findings showing that alcohol intake is a marked predictor of weaker HRV-based sleep-time recovery [50, 51]. These findings, complemented by our results, suggest that healthy dietary habits might enhance physiological recovery. On the other hand, longitudinal research is also needed to explore whether good recovery supports healthy eating.

As for eating behaviour, participants with better recovery (measured with both RMSSD and SB) scored slightly lower on intuitive eating compared with participants with weaker recovery. Lower scores on intuitive eating indicate less flexible attitudes towards eating and less eating according to body’s hunger and satiety cues [41]. The two measures of recovery, however, showed different results on the intuitive eating subscales. Better recovery measured by Stress Balance was associated with lower unconditional permission to eat, a result accompanied by a more health-promoting diet. This result is in line with studies reporting associations of higher HRV with lower unconditional permission to eat [23] and stronger dietary self-control [22, 52]. Higher parasympathetic activity during sleep measured by RMSSD associated with less eating cued by physical vs. emotional reasons but with no parallel between-group differences in diet quality. It is worth noting that in the subgroup analyses involving only those who reported working regular daytime hours, merely the associations of Stress Balance with diet quality and alcohol consumption held. While this further strengthens the notion that dietary choices are associated with the balance between stress and recovery reactions, the associations between recovery measures and eating behaviour traits remain elusive, requiring further scientific investigation.

Taken together, our results imply that in a working-age population with overweight and psychological strain, eating according to bodily cues may not necessarily associate with healthier eating behaviours. Interestingly, this result seems to contradict the theory of intuitive eating as a form of adaptive eating related to positive health outcomes and possibly higher diet quality [41, 53]. Alongside this theory, however, debate persists over the possible benefits of having regulative attitudes towards food and eating [54, 55]. Indeed, the relationship between diet quality and intuitive eating – in particular, the subscale measuring unconditional permission to eat – appears controversial. Camilleri et al. [56] reported an inverse association of unconditional permission to eat with fruit, vegetable and whole-grain product intake and a direct, weaker association with the consumption of sweet and fatty foods [56]. Similarly, in a random sample of Swiss people, unconditional permission to eat correlated moderately with poorer diet quality scores [57]. When assessing such results, considering the context is essential. Camilleri et al. [56] discussed the roles of dieting–also present in the current population–and poor health awareness as well as the obesogenic environment as probable contributors to such results [56]. Another factor that may hinder pursuing health-promoting eating habits is stress [17]. The level of perceived stress was high in the current study population, which may have acted as a notable driver for unfavourable eating behaviour [15]. Based on the evidence of stress-related unfavourable changes in food preferences, it seems that the concept of intuitive eating might show differing results depending on, e.g., the participants’ state of subjective stress. Thus, it is important to focus research on the possible mechanisms explaining this association in a stressed population with overweight.

The population of this study can be characterised by a Three-Factor Eating Questionnaire (TFEQ) score profile that implies struggling with eating behaviour [58]. All recovery groups were rather homogenous in their scores on the TFEQ subscales of emotional eating, uncontrolled eating, and cognitive restraint, whereas differences were seen in the intuitive eating scale. This may reflect the difference concerning the wording of the questionnaires: the intuitive eating scale items read along the lines of ‘I find myself eating when I’m feeling emotional…’ [41], emphasising the awareness of emotion-cued eating, whereas wording in the TFEQ is more action-directed, e.g., ‘When I feel blue, I often overeat’ [59]. Thus, better sleep-time recovery may associate with a greater awareness of the emotional drivers of eating. On the other hand, the more beneficial ratio of recovery to stress reactions seems to associate not only with a tendency to avoid eating freely whatever desired, but also with having a health-promoting diet, including low alcohol consumption. Hence, health-promoting diet may be more strongly associated with the balance between stress and recovery reactions (Stress Balance) than the mere vagally mediated recovery reflected by RMSSD.

Interestingly, total sleep time had only a weak correlation with both RMSSD and Stress Balance. This may reflect the fact that total sleep time had been calculated based on self-reported times of going to bed and waking up. Therefore, possible periods of wakefulness during this time were not accounted for.

The present study is quite unique in its focus on physiological recovery as opposed to stress, and HRV that was measured in real-life settings for several consecutive nights. Night-time activity varies less among and between individuals compared with day-time activity, which produces less noise to the data and less bias due to uncontrolled activities.

However, also several limitations apply. First, the standard methods for defining and measuring recovery are not yet established [60], which sets challenges to comparing the results of different studies; neither is it established how HRV measures ought to be utilised in nutrition science. Young and Benton [27] suggested considering HRV as a biomarker for the possible influence of food on health, but the authors also considered the possibility of a two-way interaction between eating habits and HRV. Our study, due to its cross-sectional design, allows no causality conclusions, and we have found only two randomised intervention studies that have reported causal relation between eating habits and HRV [30, 61]. Thus, further research is needed both on the effects of recovery on eating habits as well as on how nutrition might support physiological recovery.

Also, the validity and reliability of the questionnaires should be considered. The 48-h dietary recall captured days between Sunday and Thursday. Therefore, stress-related eating occurring outside the measurement days may have been involved. Neither can the possibilities of recall bias or underreporting be ruled out. Second, as noted by Tylka [41], the Intuitive eating subscales measuring eating for physical rather than emotional reasons as well as unconditional permission to eat are worded to measure the absence of emotional and conditional eating, respectively, resulting in possible validity issues [41]. As some of the subscales (IES *Unconditional permission to eat*; TFEQ-R18 *Cognitive restraint*; ecSI 2.0™ *Eating attitudes*, *Food acceptance,* and *Internal regulation*) showed less than desirable reliability with Cronbach’s α < 0.7, both quantitative and qualitative research into the reliability of eating behaviour scales in different populations is advisable.

The study population consisted of working-age adults with psychological distress and overweight, which limits the generalisability of our results. Nevertheless, the present study concerns a high-risk group for developing negative health outcomes that may impede work ability, which warrants the importance of further research in this population. Also, as in many previous studies, the majority of the participants (83%) were female. To the best of our knowledge, only one study so far has addressed the effects of gender: findings by George et al. [62] suggest that vegetarianism and being female have a positive interaction effect on HRV, which further underlines the need to investigate the role of gender in the context of eating habits and recovery.

Lastly, the Elixir study had not been designed for the present investigation, allowing for limited controlling for confounding factors. Taking all limitations into account, the small magnitudes of the effect sizes of recovery were to be expected. HRV is affected by several factors, such as age, stress, and physical activity level [63], so aiming to control for confounding factors in future intervention studies is important to get as valid results as possible.

To conclude, our results suggest that among working-age people with perceived stress and overweight, better sleep-time recovery is associated with more health-promoting diet and possibly less intuitive eating. In future lifestyle interventions and in clinical practice, it is important to address sleep-time recovery as one factor related to eating habits. For further progress in both occupational health and nutrition sciences, we call for randomised controlled interventions that aim at maximising sleep-time recovery and investigate its effects on eating habits. Also dietary interventions investigating subsequent effects on sleep-time recovery are needed.

## Supplementary Information

**Additional file 1: Table 1.** The main effects of recovery group for all eating behaviour and diet quality variables.

## Data Availability

The dataset is not publicly available due to considerations of data protection.

## Abbreviations

ANS: Autonomic nervous system
AUDIT-C: Alcohol Use Disorders Identification Test Consumption
ecSI 2.0™: Preliminary Finnish translation of Satter Eating Competence Inventory 2.0
GHQ-12: 12-item General Health Questionnaire
HRV: Heart rate variability
HTAS: Health and Taste Attitude Scales
IDQ: Index of Diet Quality
IES: Intuitive Eating Scale
PSS: Perceived Stress Scale
RMSSD: Root Mean Square of the Successive Differences
TFEQ-R18: The 18-item Three-Factor Eating Questionnaire
WAI: Work Ability Index

## References

[1] Suvisaari J, Viertiö S, Solin P, Partonen T, Koponen P, Borodulin K, Lundqvist A, Sääksjärvi K, Koskinen S (2018). Mental health. Health, functional capacity and welfare in Finland - FinHealth 2017 study.

[2] Lundqvist A, Männistö S, Jousilahti P, Kaartinen N, Mäki P, Borodulin K, Koponen P, Borodulin K, Lundqvist A, Sääksjärvi K, Koskinen S (2018). Obesity. Health, functional capacity and welfare in Finland - FinHealth 2017 study.

[3] Partonen T, Lundqvist A, Wennman H, Borodulin K, Koponen P, Borodulin K, Lundqvist A, Sääksjärvi K, Koskinen S (2018). Sleep. Health, functional capacity and welfare in Finland - FinHealth 2017 study.

[4] Theorell T. Anabolism and catabolism - antagonistic partners in stress and strain. Scand J Work Environ Health. 2008;(6):136–43.

[5] Uusitalo A, Mets T, Martinmäki K, Mauno S, Kinnunen U, Rusko H (2011). Heart rate variability related to effort at work. Appl Ergon.

[6] Lindholm H (2013). Physiological determinants and assessment of stress and recovery among media workers [doctoral dissertation]: Finnish Institute of Occupational Health.

[7] Pantzar M, Ruckenstein M, Mustonen V (2017). Social rhythms of the heart. Health Sociol Rev.

[8] Kinnunen U, Mauno S, Siltaloppi M (2010). Job insecurity, recovery and well-being at work: recovery experiences as moderators. Econ Ind Democr.

[9] McEwen BS (1998). Protective and damaging effects of stress mediators. N Engl J Med.

[10] Teisala T, Mutikainen S, Tolvanen A, Rottensteiner M, Leskinen T, Kaprio J, Kolehmainen M, Rusko H, Kujala UM (2014). Associations of physical activity, fitness, and body composition with heart rate variability-based indicators of stress and recovery on workdays: a cross-sectional study. J Occup Med Toxicol..

[11] Föhr T, Tolvanen A, Myllymäki T, Järvelä-Reijonen E, Rantala S, Korpela R, Peuhkuri K, Kolehmainen M, Puttonen S, Lappalainen R, Rusko H, Kujala UM (2015). Subjective stress, objective heart rate variability-based stress, and recovery on workdays among overweight and psychologically distressed individuals: a cross-sectional study. J Occup Med Toxicol.

[12] Task Force of the European Society of Cardiology and the North American Society of Pacing and Electrophysiology (1996). Heart rate variability: standards of measurement, physiological interpretation and clinical use. Circulation..

[13] Acharya UR, Joseph KP, Kannathal N, Lim CM, Suri JS (2006). Heart rate variability: a review. Med Biol Eng Comput.

[14] Drew RC, Sinoway LI, Robertson D, Biaggioni I, Burnstock G, Low PA, Paton JFR (2012). Autonomic control of the heart. Primer on the autonomic nervous system.

[15] Järvelä-Reijonen E, Karhunen L, Sairanen E, Rantala S, Laitinen J, Puttonen S, Peuhkuri K, Hallikainen M, Juvonen K, Myllymäki T, Föhr T, Pihlajamäki J, Korpela R, Ermes M, Lappalainen R, Kolehmainen M (2016). High perceived stress is associated with unfavorable eating behavior in overweight and obese Finns of working age. Appetite..

[16] Nevanperä NJ, Hopsu L, Kuosma E, Ukkola O, Uitti J, Laitinen JH (2012). Occupational burnout, eating behavior, and weight among working women. Am J Clin Nutr.

[17] Groesz LM, McCoy S, Carl J, Saslow L, Stewart J, Adler N, Laraia B, Epel E (2012). What is eating you? Stress and the drive to eat. Appetite..

[18] Jääskeläinen A, Nevanperä N, Remes J, Rahkonen F, Järvelin MR, Laitinen J (2014). Stress-related eating, obesity and associated behavioural traits in adolescents: a prospective population-based cohort study. BMC Public Health.

[19] Hemiö K, Lindström J, Peltonen M, Härmä M, Viitasalo K, Puttonen S (2020). High need for recovery from work and sleep problems are associated with workers’ unhealthy dietary habits. Public Health Nutr.

[20] Ranzenhofer LM, Engel SG, Crosby RD, Haigney M, Anderson M, McCaffery JM (2016). Real-time assessment of heart rate variability and loss of control eating in adolescent girls: a pilot study. Int J Eat Disord.

[21] Young HA, Cousins AL, Watkins HT, Benton D (2017). Is the link between depressed mood and heart rate variability explained by disinhibited eating and diet?. Biol Psychol.

[22] Maier SU, Hare TA (2017). Higher heart-rate variability is associated with ventromedial prefrontal cortex activity and increased resistance to temptation in dietary self-control challenges. J Neurosci.

[23] Peschel SKV, Tylka TL, Williams DP, Kaess M, Thayer JF, Koenig J (2018). Is intuitive eating related to resting state vagal activity?. Auton Neurosci.

[24] Meule A, Vögele C, Kübler A (2012). Restrained eating is related to accelerated reaction to high caloric foods and cardiac autonomic dysregulation. Appetite..

[25] Finch LE, Cummings JR, Tomiyama AJ (2019). Cookie or clementine? Psychophysiological stress reactivity and recovery after eating healthy and unhealthy comfort foods. Psychoneuroendocrinology..

[26] Udo T, Weinberger AH, Grilo CM, Brownell KD, DiLeone RJ, Lampert R, Matlin SL, Yanagisawa K, McKee S (2014). Heightened vagal activity during high-calorie food presentation in obese compared with non-obese individuals--results of a pilot study. Obes Res Clin Pract.

[27] Young HA, Benton D (2018). Heart-rate variability: a biomarker to study the influence of nutrition on physiological and psychological health?. Behav Pharmacol.

[28] Reginato E, Azzolina D, Folino F, Valentini R, Bendinelli C, Gafare CE, Cainelli E, Vedovelli L, Iliceto S, Gregori D, Lorenzoni G (2020). Dietary and lifestyle patterns are associated with heart rate variability. J Clin Med.

[29] Dai J, Lampert R, Wilson PW, Goldberg J, Ziegler TR, Vaccarino V (2010). Mediterranean dietary pattern is associated with improved cardiac autonomic function among middle-aged men: a twin study. Circ Cardiovasc Qual Outcomes.

[30] Jaatinen N, Korpela R, Poussa T, Turpeinen A, Mustonen S, Merilahti J, Peuhkuri K (2014). Effects of daily intake of yoghurt enriched with bioactive components on chronic stress responses: a double-blinded randomized controlled trial. Int J Food Sci Nutr.

[31] Gissoni NB, dos Santos Quaresma MVL (2020). Short sleep duration and food intake: an overview and analysis of the influence of the homeostatic and hedonic system. Nutrire..

[32] Pérez-Fuentes MDC, Molero Jurado MDM, Barragán Martín AB, Martos Martínez Á, Gázquez Linares JJ (2019). Association with the quality of sleep and the mediating role of eating on self-esteem in healthcare personnel. Nutrients..

[33] Park SK, Tucker KL, O'Neill MS, Sparrow D, Vokonas PS, Hu H (2009). Fruit, vegetable, and fish consumption and heart rate variability: the veterans administration normative aging study. Am J Clin Nutr.

[34] Lappalainen R, Sairanen E, Järvelä E, Rantala S, Korpela R, Puttonen S (2014). The effectiveness and applicability of different lifestyle interventions for enhancing wellbeing: the study design for a randomized controlled trial for persons with metabolic syndrome risk factors and psychological distress. BMC Public Health.

[35] Goldberg D (1972). The detection of psychiatric illness by questionnaire. Maudsley monograph no. 21.

[36] Kelsey RM, Soderlund K, Arthur CM (2004). Cardiovascular reactivity and adaptation to recurrent psychological stress: replication and extension. Psychophysiology..

[37] Firstbeat Technologies Ltd. Stress and recovery analysis method based on 24-hour heart rate variability. White paper [Internet]. Firstbeat Technologies Ltd; 2014. https://www.firstbeat.com/en/science-and-physiology/white-papers-and-publications/. Accessed 15 Nov 2016.

[38] Saalasti S, Seppänen M, Kuusela A. Artefact correction for heart beat interval data. Advanced methods for processing bioelectrical signals. In: Proceedings of the ProBisi Meeting, vol. 2004. Jyväskylä; 2004. https://assets.firstbeat.com/firstbeat/uploads/2015/11/saalasti_et_al_probisi_2004_congress.pdf. Accessed 21 April 2017.

[39] Shaffer F, Ginsberg JP (2017). An overview of heart rate variability metrics and norms. Front Public Health.

[40] Penttilä J, Helminen A, Jartti T, Kuusela T, Huikuri HV, Tulppo MP, Coffeng R, Scheinin H (2001). Time domain, geometrical and frequency domain analysis of cardiac vagal outflow: effects of various respiratory patterns. Clin Physiol.

[41] Tylka T (2006). Development and psychometric evaluation of a measure of intuitive eating. J Couns Psychol.

[42] Karlsson J, Persson LO, Sjöström L, Sullivan M (2000). Psychometric properties and factor structure of the three-factor eating questionnaire (TFEQ) in obese men and women. Results from the Swedish obese subjects (SOS) study. Int J Obes Relat Metab Disord.

[43] Roininen K, Lähteenmäki L, Tuorila H (1999). Quantification of consumer attitudes to health and hedonic characteristics of foods. Appetite..

[44] Lohse B (2015). The Satter eating competence inventory for Low-income persons is a valid measure of eating competence for persons of higher socioeconomic position. Appetite..

[45] Leppälä J, Lagström H, Kaljonen A, Laitinen K (2010). Construction and evaluation of a self-contained index for assessment of diet quality. Scand J Public Health.

[46] Bush K, Kivlahan DR, McDonell MB, Fihn SD, Bradley KA (1998). The AUDIT alcohol consumption questions (AUDIT-C): an effective brief screening test for problem drinking. Ambulatory care quality improvement project (ACQUIP). Alcohol use disorders identification test. Arch Intern Med.

[47] Cohen S, Kamarck T, Mermelstein R (1983). A global measure of perceived stress. J Health Soc Behav.

[48] Tuomi K, Ilmarinen J, Jahkola A, Katajarinne L, Tulkki A (1998). Work ability index. Occupational health care 19. 2nd revised ed.

[49] Kogan A, Gruber J, Shallcross AJ, Ford BQ, Mauss IB (2013). Too much of a good thing? Cardiac vagal tone’s nonlinear relationship with well-being. Emotion..

[50] Pietilä J, Helander E, Myllymäki T, Korhonen I, Jimison H, Pavel M (2015). Exploratory analysis of associations between individual lifestyles and heart rate variability -based recovery during sleep. Conf Proc IEEE Eng Med Biol Soc.

[51] Pietilä J, Helander E, Korhonen I, Myllymäki T, Kujala UM, Lindholm H (2018). Acute effect of alcohol intake on cardiovascular autonomic regulation during the first hours of sleep in a large real-world sample of Finnish employees: observational study. JMIR Ment Health.

[52] Segerstrom SC, Nes LS (2007). Heart rate variability reflects self-regulatory strength, effort, and fatigue. Psychol Sci.

[53] Van Dyke N, Drinkwater EJ (2014). Relationships between intuitive eating and health indicators: literature review. Public Health Nutr.

[54] Dohle S, Diel K, Hofmann W (2018). Executive functions and the self-regulation of eating behavior: a review. Appetite..

[55] Johnson F, Pratt M, Wardle J (2012). Dietary restraint and self-regulation in eating behavior. Int J Obes.

[56] Camilleri GM, Méjean C, Bellisle F, Andreeva VA, Kesse-Guyot E, Hercberg S, Péneau S (2017). Intuitive eating dimensions were differently associated with food intake in the general population-based NutriNet-Santé study. J Nutr.

[57] Horwath C, Hagmann D, Hartmann C (2019). Intuitive eating and food intake in men and women: results from the Swiss food panel study. Appetite..

[58] Pentikäinen S, Arvola A, Karhunen L, Pennanen K (2018). Easy-going, rational, susceptible and struggling eaters: a segmentation study based on eating behaviour tendencies. Appetite..

[59] de Lauzon B, Romon M, Deschamps V, Lafay L, Borys JM, Karlsson J, Ducimetière P, Charles MA, Fleurbaix Laventie Ville Sante Study Group (2004). The three-factor eating questionnaire-R18 is able to distinguish among different eating patterns in a general population. J Nutr.

[60] Verbeek J, Ruotsalainen J, Laitinen J, Korkiakangas E, Lusa S, Mänttäri S, Oksanen T (2019). Interventions to enhance recovery in healthy workers; a scoping review. Occup Med (Lond).

[61] Hansen AL, Dahl L, Olson G, Thornton D, Graff IE, Froyland L (2014). Fish consumption, sleep, daily functioning, and heart rate variability. J Clin Sleep Med.

[62] George K, Immaculate Joy S, Thomas NS, Balamurali R, Baskaran K (2021). Gender-based vegetarian and nonvegetarian dietary impact on cardiac autonomic function of heart rate variability. J Am Coll Nutr.

[63] Fatisson J, Oswald V, Lalonde F (2016). Influence diagram of physiological and environmental factors affecting heart rate variability: an extended literature overview. Heart Int.

