# Supplementary material for: Sleep-time physiological recovery is associated with eating habits in distressed working-age Finns with overweight: secondary analysis of a randomised controlled trial
Source: J Occup Med Toxicol. 2021 Jun 28;16:23. doi: 10.1186/s12995-021-00310-6 (PMC8237494; doi:10.1186/s12995-021-00310-6)
Supplement: Supplementary file 1 — Additional file 1: Table 1. The main effects of recovery group for all eating behaviour and diet quality variables. [file 12995_2021_310_MOESM1_ESM.docx]

**Table 1.** Eating behaviour questionnaire scores among the RMSSD tertiles (N=252). [RMSSD=Root Mean Square of the Successive Differences; IES=Intuitive Eating Scale; TFEQ-R18=The Three-Factor Eating Questionnaire-R18; HTAS=Health and Taste Attitude Scales; ecSI 2.0™=preliminary Finnish translation of Satter Eating Competence Inventory 2.0.]

|  |  | Weakest recovery, RMSSD  (*n*=84) | |  | Average recovery, RMSSD  (*n*=84) | |  | Best  recovery, RMSSD  (*n*=84) | |  |  |
| --- | --- | --- | --- | --- | --- | --- | --- | --- | --- | --- | --- |
| Eating behaviour measure | Possible score range | Mean (SD) | |  | Mean (SD) | |  | Mean (SD) | | P^a^ | P^b^ |
| IES total score | 1 – 5 | 3.1 (0.5)^1^ | |  | 2.9 (0.5) | |  | 2.9 (0.5)^2^ | | **0.019** | **0.013** |
| Unconditional Permission to Eat | 1 – 5 | 3.2 (0.6) | |  | 3.2 (0.6) | |  | 3.0 (0.6) | | 0.198 | 0.240 |
| Eating for Physical Rather Than Emotional Reasons | 1 – 5 | 2.7 (0.9)^1^ | |  | 2.4 (0.8) | |  | 2.3 (0.7)^2^ | | **0.010** | **0.004** |
| Reliance on Internal Hunger/Satiety Cues | 1 – 5 | 3.2 (0.7) | |  | 3.1 (0.6) | |  | 3.2 (0.6) | | 0.474 | 0.415 |
| TFEQ-R18 |  |  |  |  |  |  |  |  |  |  |  |
| Cognitive Restraint | 0 – 100 | 44.3 (15.3) | |  | 41.0 (16.3) | |  | 45.5 (14.6) | | 0.146 | 0.152 |
| Uncontrolled Eating | 0 – 100 | 45.9 (19.0) | |  | 48.6 (19.9) | |  | 52.1 (17.4) | | 0.097 | 0.126 |
| Emotional Eating | 0 – 100 | 57.9 (30.2) | |  | 61.0 (26.9) | |  | 61.1 (24.1) | | 0.645 | 0.473 |
| HTAS |  |  |  |  |  |  |  |  |  |  |  |
| Pleasure | 1 – 7 | 4.9 (1.1) | |  | 4.7 (1.1) | |  | 4.6 (0.9) | | 0.346 | 0.361 |
| Using Food as a Reward | 1 – 7 | 4.3 (1.3) | |  | 4.5 (1.2) | |  | 4.5 (1.0) | | 0.346 | 0.307 |
| ecSI 2.0™ total score | 0 – 48 | 26.5 (5.8) | |  | 25.7 (5.6) | |  | 26.7 (6.5) | | 0.505 | 0.351 |
| Eating Attitudes | 0 – 15 | 10.0 (2.2) | |  | 9.8 (2.4) | |  | 9.9 (2.5) | | 0.854 | 0.837 |
| Food Acceptance | 0 – 9 | 4.9 (1.8) | |  | 4.9 (1.8) | |  | 5.2 (2.1) | | 0.520 | 0.422 |
| Internal Regulation | 0 – 9 | 5.0 (1.8) | |  | 4.7 (1.6) | |  | 5.0 (1.9) | | 0.429 | 0.307 |
| Contextual Skills | 0 – 15 | 6.54 (3.0) | |  | 6.3 (3.0) | |  | 6.6 (3.0) | | 0.721 | 0.611 |

Means are unadjusted means.

^a^Recovery group main effect (three-way ANOVA with RMSSD recovery group, study center, and starting time of the study as independent variables). According to Šidák-corrected post hoc comparisons, value marked with ^1^ is significantly higher than value marked with ^2^.

^b^Recovery group main effect (ANCOVA with RMSSD recovery group, study center, starting time of the study, gender, and BMI as independent variables).

**Table 2.** Eating behaviour questionnaire scores among the Stress Balance groups (N=252). [SB=Stress Balance; IQR=interquartile range; IES=Intuitive Eating Scale; TFEQ-R18=The Three-Factor Eating Questionnaire-R18; HTAS=Health and Taste Attitude Scales; ecSI 2.0™=preliminary Finnish translation of Satter Eating Competence Inventory 2.0.]

|  |  | Weak recovery, SB  (*n*=59) | |  | Moderate recovery, SB  (*n*=87) | |  | Good recovery, SB  (*n=*106) | |  |  |
| --- | --- | --- | --- | --- | --- | --- | --- | --- | --- | --- | --- |
| Eating behaviour measure | Possible score range | Mean (SD) | Median (IQR) |  | Mean (SD) | Median (IQR) |  | Mean (SD) | Median (IQR) | P^a^ | P^b^ |
| IES total score | 1 – 5 | 3.0 (0.5) |  |  | 3.0 (0.5) |  |  | 2.9 (0.5) |  | 0.114 | **0.039** |
| Unconditional Permission to Eat | 1 – 5 | 3.3 (0.7)^1^ |  |  | 3.2 (0.6) |  |  | 3.0 (0.6)^2^ |  | **0.008** | **0.002** |
| Eating for Physical Rather Than Emotional Reasons^c^ | 1 – 5 |  | 2.3 (1.8-2.8) |  |  | 2.5 (2.0-3.2) |  |  | 2.3 (2.0-2.7) | 0.320 | 0.232 |
| Reliance on Internal Hunger/Satiety Cues^d^ | 1 – 5 |  | 3.2 (2.7-3.5) |  |  | 3.2 (2.7-3.5) |  |  | 3.3 (2.8-3.7) | 0.514 | 0.685 |
| TFEQ-R18 |  |  |  |  |  |  |  |  |  |  |  |
| Cognitive Restraint | 0 – 100 | 42.6 (17.3) |  |  | 41.2 (14.1) |  |  | 46.2 (15.2) |  | 0.089 | 0.082 |
| Uncontrolled Eating | 0 – 100 | 46.5 (19.9) |  |  | 50.2 (17.9) |  |  | 49.1 (19.2) |  | 0.368 | 0.242 |
| Emotional Eating^e^ | 0 – 100 |  | 66.7 (44.4-77.8) |  |  | 66.7 (33.3-77.8) |  |  | 66.7 (44.4-77.8) | 0.482 | 0.392 |
| HTAS |  |  |  |  |  |  |  |  |  |  |  |
| Pleasure | 1 – 7 | 4.9 (1.0) |  |  | 4.7 (1.0) |  |  | 4.7 (1.0) |  | 0.383 | 0.396 |
| Using Food as a Reward^e^ | 1 – 7 |  | 4.7 (3.8-5.3) |  |  | 4.3 (3.5-5.3) |  |  | 4.7 (4.0-5.2) | 0.534 | 0.501 |
| ecSI 2.0™ total score | 0 – 48 | 26.0 (6.3) |  |  | 25.7 (5.7) |  |  | 26.9 (6.1) |  | 0.431 | 0.445 |
| Eating Attitudes | 0 – 15 | 10.3 (2.4) |  |  | 9.7 (2.4) |  |  | 9.9 (2.3) |  | 0.356 | 0.288 |
| Food Acceptance | 0 – 9 | 4.5 (1.8) |  |  | 4.9 (1.9) |  |  | 5.3 (1.9) |  | 0.116 | 0.150 |
| Internal Regulation | 0 – 9 | 5.0 (1.9) |  |  | 4.6 (2.0) |  |  | 5.1 (1.5) |  | 0.071 | 0.063 |
| Contextual Skills | 0 – 15 | 6.2 (2.9) |  |  | 6.5 (2.9) |  |  | 6.6 (3.1) |  | 0.735 | 0.831 |

Values are unadjusted means and SDs for variables with normally distributed residuals and median and IQR for other variables.

^a^Recovery group main effect (three-way ANOVA with SB recovery group, study center, and starting time of the study as independent variables). According to Šidák-corrected post hoc comparisons, value marked with ^1^ is significantly higher than value marked with ^2^.

^b^Recovery group main effect (ANCOVA with SB recovery group, study center, starting time of the study, gender, and BMI as independent variables).

^c^Data transformed (LN) to achieve residual normality.

^d^Data transformed (LN reverse) to achieve residual normality.

^e^Data transformed (square root reverse) to achieve residual normality.

**Table 3.** Diet quality measures among the RMSSD tertiles (N=252). [RMSSD=Root Mean Square of the Successive Differences; IQR=interquartile range; IDQ=total score of Index of Diet Quality questionnaire (possible score range 0–15); AUDIT-C=total score of three alcohol consumption questions from the Alcohol Use Disorders Identification Test (possible score range 0–12); E%=percentage of energy.]

|  |  | Weakest recovery, RMSSD  (*n*=84) | |  | Average recovery, RMSSD  (*n=*84) | |  | Best  recovery, RMSSD  (*n*=84) | |  |  |
| --- | --- | --- | --- | --- | --- | --- | --- | --- | --- | --- | --- |
| Dietary measure | Range | Mean (SD) | Median (IQR) |  | Mean (SD) | Median (IQR) |  | Mean (SD) | Median (IQR) | P^a^ | P^b^ |
| IDQ^c^ | 5–15 | 9.95 (2.1) |  |  | 10.12 (2.0) |  |  | 10.57 (2.1) |  | 0.183 | 0.175 |
| AUDIT-C | 0–11 | 4.2 (2.5) |  |  | 3.4 (2.1) |  |  | 3.9 (2.3) |  | 0.061 | **0.029** |
| Energy (kcal) | 666.1–4686.5 | 1919.0 (527.4) |  |  | 1951.8 (508.4) |  |  | 2083.1 (587.0) |  | 0.126 | 0.117 |
| Protein (E%) | 8.6–29.0 | 17.2 (4.2) |  |  | 17.1 (3.6) |  |  | 17.8 (3.9) |  | 0.458 | 0.517 |
| Fat (E%) | 15.9–65.3 | 37.5 (8.6) |  |  | 37.3 (7.7) |  |  | 37.7 (7.5) |  | 0.918 | 0.943 |
| SFA (E%) | 5.1–26.7 | 13.7 (3.3) |  |  | 13.3 (3.7) |  |  | 13.8 (4.0) |  | 0.627 | 0.686 |
| MUFA (E%) | 3.3–23.7 | 13.0 (3.8) |  |  | 12.8 (3.3) |  |  | 13.0 (2.9) |  | 0.891 | 0.903 |
| PUFA (E%)^d^ | 1.8–18.4 |  | 5.6 (4.7-7.2) |  |  | 6.4 (4.7-7.9) |  |  | 6.3 (5.3-7.3) | 0.636 | 0.666 |
| Carbohydrates (E%) | 10.6–67.3 | 40.7 (7.6) |  |  | 42.1 (7.5) |  |  | 40.7 (7.7) |  | 0.357 | 0.357 |
| Sucrose (E%)^d^ | 0.9–42.5 |  | 8.9 (6.5-10.9) |  |  | 8.9 (6.2-12.5) |  |  | 7.7 (5.2-10.9) | 0.338 | 0.378 |
| Fibre (g/MJ) | 0.9–6.3 | 2.9 (1.0) |  |  | 2.9 (1.1) |  |  | 2.7 (0.9) |  | 0.392 | 0.460 |

Values are unadjusted means and SDs for variables with normally distributed residuals and median and IQR for other variables.

^a^Recovery group main effect (three-way ANOVA with RMSSD recovery group, study center, and starting time of the study as independent variables).

^b^Recovery group main effect (ANCOVA with RMSSD recovery group, study center, starting time of the study, gender, and BMI as independent variables). According to Šidák-corrected post hoc comparisons, value marked with ^1^ is significantly higher than value marked with ^2^.

^c^Best recovery *n*=83.

^d^LN transformed variable.

**Table 4.** Diet quality measures among the Stress Balance groups (N=252). [SB=Stress Balance; IQR=interquartile range; IDQ=total score of Index of Diet Quality questionnaire (possible score range 0–15); AUDIT-C=total score of three alcohol consumption questions from the Alcohol Use Disorders Identification Test (possible score range 0–12); E%=percentage of energy.]

|  |  | Weak recovery, SB  (*n*=59) | |  | Moderate recovery, SB  (*n*=87) | |  | Good  recovery, SB  (*n*=106) | |  |  |
| --- | --- | --- | --- | --- | --- | --- | --- | --- | --- | --- | --- |
| Dietary measure | Range | Mean (SD) | Median (IQR) |  | Mean (SD) | Median (IQR) |  | Mean (SD) | Median (IQR) | P^a^ | P^b^ |
| IDQ^c^ | 5–15 | 9.3 (2.2)^2^ |  |  | 10.2 (2.0)^1^ |  |  | 10.7 (1.9)^1^ |  | **0.001** | **0.001** |
| AUDIT-C | 0–11 | 5.2 (2.7)^1^ |  |  | 3.7 (2.1)^2^ |  |  | 3.3 (2.1)^2^ |  | **<0.001** | **<0.001** |
| Energy (kcal) | 666.1–4686.5 | 1971.4 (584.8) |  |  | 2031.1 (527.1) |  |  | 1953.9 (537.8) |  | 0.571 | 0.498 |
| Protein (E%)^d^ | 8.6–29.0 |  | 16.5 (14.2-20.2) |  |  | 16.5 (14.2-19.6) |  |  | 17.3 (14.9-19.9) | 0.545 | 0.507 |
| Fat (E%) | 15.9–65.3 | 38.7 (8.2) |  |  | 37.0 (6.8) |  |  | 37.2 (8.6) |  | 0.519 | 0.567 |
| SFA (E%) | 5.1–26.7 | 14.2 (3.8) |  |  | 13.5 (3.3) |  |  | 13.3 (3.8) |  | 0.429 | 0.486 |
| MUFA (E%) | 3.3–23.7 | 13.3 (3.5) |  |  | 12.6 (3.1) |  |  | 12.9 (3.4) |  | 0.557 | 0.589 |
| PUFA (E%)^d^ | 1.77–18.4 |  | 6.1 (4.8-8.0) |  |  | 6.1 (4.8-7.2) |  |  | 6.2 (6.1-11.5) | 0.824 | 0.892 |
| Carbohydrates (E%) | 10.6–67.3 | 39.8 (6.9) |  |  | 41.3 (6.8) |  |  | 41.8 (8.4) |  | 0.292 | 0.334 |
| Sucrose (E%)^d^ | 0.9–42.5 |  | 8.1 (5.8-10.7) |  |  | 8.6 (6.2-11.5) |  |  | 8.9 (6.1-11.5) | 0.881 | 0.926 |
| Fibre (g/MJ)^d^ | 0.9–6.3 |  | 2.6 (1.7-3.1)^2^ |  |  | 2.5 (2.1-3.5) |  |  | 2.8 (2.3-3.6)^1^ | **0.028** | **0.029** |

Values are unadjusted means and SDs for variables with normally distributed residuals and median and IQR for other variables.

^a^Recovery group main effect (three-way ANOVA with SB recovery group, study center, and starting time of the study as independent variables). According to Šidák-corrected post hoc comparisons, value marked with ^1^ is significantly higher than value marked with ^2^.

^b^Recovery group main effect (ANCOVA with SB recovery group, study center, starting time of the study, gender, and BMI as independent variables).

^c^Weak recovery *n=*58.

^d^Data transformed (LN) to achieve residual normality.
